# Artificial intelligence-based quantification of pulmonary HRCT (AIqpHRCT) for the evaluation of interstitial lung disease in patients with inflammatory rheumatic diseases

**DOI:** 10.1007/s00296-024-05715-0

**Published:** 2024-09-09

**Authors:** Tobias Hoffmann, Ulf Teichgräber, Bianca Lassen-Schmidt, Diane Renz, Luis Benedict Brüheim, Martin Krämer, Peter Oelzner, Joachim Böttcher, Felix Güttler, Gunter Wolf, Alexander Pfeil

**Affiliations:** 1 Department of Internal Medicine III, Jena University Hospital – Friedrich Schiller University Jena, Jena, Germany; 2 Institute of Diagnostic and Interventional Radiology, Jena University Hospital – Friedrich Schiller University Jena, Jena, Germany; 3 Fraunhofer Institute for Digital Medicine MEVIS, Bremen, Germany; 4 Institute of Diagnostic and Interventional Radiology, Department of Pediatric Radiology, Hannover Medical School, Hannover, Germany; 5 Department of Internal Medicine III, Center of Rheumatology, Jena University Hospital – Friedrich Schiller University Jena, Am Klinikum 1, 07747 Jena, Germany

**Keywords:** Interstitial lung disease, Inflammatory rheumatic diseases, High-resolution computed tomography, Artificial intelligence-based quantification of pulmonary, Quantification

## Abstract

High-resolution computed tomography (HRCT) is important for diagnosing interstitial lung disease (ILD) in inflammatory rheumatic disease (IRD) patients. However, visual ILD assessment via HRCT often has high inter-reader variability. Artificial intelligence (AI)-based techniques for quantitative image analysis promise more accurate diagnostic and prognostic information. This study evaluated the reliability of artificial intelligence-based quantification of pulmonary HRCT (AIqpHRCT) in IRD-ILD patients and verified IRD-ILD quantification using AIqpHRCT in the clinical setting. Reproducibility of AIqpHRCT was verified for each typical HRCT pattern (ground-glass opacity [GGO], non-specific interstitial pneumonia [NSIP], usual interstitial pneumonia [UIP], granuloma). Additional, 50 HRCT datasets from 50 IRD-ILD patients using AIqpHRCT were analysed and correlated with clinical data and pulmonary lung function parameters. AIqpHRCT presented 100% agreement (coefficient of variation = 0.00%, intraclass correlation coefficient = 1.000) regarding the detection of the different HRCT pattern. Furthermore, AIqpHRCT data showed an increase of ILD from 10.7 ± 28.3% (median = 1.3%) in GGO to 18.9 ± 12.4% (median = 18.0%) in UIP pattern. The extent of fibrosis negatively correlated with FVC (ρ=-0.501), TLC (ρ=-0.622), and DLCO (ρ=-0.693) (*p* < 0.001). GGO measured by AIqpHRCT also significant negatively correlated with DLCO (ρ=-0.699), TLC (ρ=-0.580) and FVC (ρ=-0.423). For the first time, the study demonstrates that AIpqHRCT provides a highly reliable method for quantifying lung parenchymal changes in HRCT images of IRD-ILD patients. Further, the AIqpHRCT method revealed significant correlations between the extent of ILD and lung function parameters. This highlights the potential of AIpqHRCT in enhancing the accuracy of ILD diagnosis and prognosis in clinical settings, ultimately improving patient management and outcomes.

**Supplementary Information:**

The online version contains supplementary material available at 10.1007/s00296-024-05715-0.

## Introduction

Lung involvement is the most common and serious organ manifestation in patients with inflammatory rheumatic disease (IRD). The type of pulmonary complications can differ, but the most typical manifestation is interstitial lung disease (ILD-IRD). IRD with the highest likelihood of pulmonary involvement are connective tissue diseases (CTD; systemic sclerosis [SSc] 30.8%, mixed connective tissue disease [MCTD] 25.0%, Sjogren’s syndrome 20.0%, systemic lupus erythematosus [SLE] 12.5%,), vasculitis (microscopic polyangiitis [MPA] 83.3%, granulomatosis with polyangiitis [GPA] 80.0%, eosinophilic granulomatosis with polyangiitis [EGPA] 66.7%), and myositis (33.3%, dermatomyositis 100.0%) [1].

Given the significant morbidity and mortality, early diagnosis and severity assessment of ILD-IRD is essential, especially in the light of modern antifibrotic treatment options. In most cases, the diagnosis of IRD-ILD is made based on the combination of clinical symptoms, physical examination, non-invasive diagnostic tools (e.g. pulmonary function test [PFT]), and high-resolution computed tomography (HRCT) [2].

HRCT is a key component in this multidisciplinary approach, adding important information that cannot be determined from medical history and other diagnostic tests such as PFT [1–5]. The most common ILD patterns in HRCT include ground-glass opacity (GGO), non-specific interstitial pneumonia (NSIP), usual interstitial pneumonia (UIP), and granuloma [2, 5–8]. Although granuloma are not a typical manifestation of fibrosing ILD, they are an important and frequent pulmonary manifestation of IRD, especially in small vessel vasculitis [2].

As the diagnosis of ILD is usually made without a lung biopsy, the reliable detection of these HRCT features is essential. Currently, visual assessment by an experienced clinician is the gold standard for assessing IRD-ILD using HRCT [9]. However, several studies have shown substantial inter-observer variations in the visual (qualitative) evaluation of ILD by HRCT even among experienced thoracic radiologists [8, 10–12]. Furthermore, there is no validated and established scoring system to verify and quantify IRD-ILD. Visual scores are inconsistent and relatively irreproducible, and even experienced chest radiologists may struggle with the differential diagnosis. Furthermore, the importance of reliable measurement of fibrosis on HRCT is underlined by data showing poorer outcomes in patients with SSc with > 2% progression of fibrosis [13]. In this context, qualitative visual assessment can indicate whether lung parenchymal changes are present or not, but reliable quantification cannot be performed visually.

The use of artificial intelligence (AI) systems can be helpful to overcome these obstacles, allowing a reliable quantification of the extent of ILD features in HRCT [14]. AI-based methods for the quantification of ILD on HRCT (artificial intelligence-based quantification of pulmonary HRCT - AIqpHRCT) could provide a rapid, objective and quantitative measure of the extent of disease that is independent of the expertise of the reader [15]. To date, especially in IRD-ILD, traditional methods such as histogram analysis or pattern/texture-based analysis have been mainly used to quantify parenchymal findings in ILD (25–31). The number of studies using computer-assisted analysis of IRD-ILD has been limited, and has mainly focused on patients with SSc (27–30), supplemented by data on idiopathic pulmonary fibrosis (32–34). To the best of our knowledge, no study has yet been published that concentrates on an AI approach to the quantitative analysis of the lungs from HRCT in patients with IRD-ILD.

Such an AI tool is SATORI (Segmentation and Annotation TOol for Radiomics and Deep LearnIng), a browser-based platform for curating medical data, developed by the Fraunhofer Institute for Digital Medicine Mevis, Bremen/Germany. The advanced SATORI optimization for lung image analysis allows efficient automatic segmentation of the lungs, lung lobes, bronchi, and blood vessels as well as pathologies on HRCT data and provides a workflow for quantitative lung parenchyma analysis [15, 16]. Currently, SATORI is used in more than 15 research projects, the largest being RACOON (RAdiological COOperative Network) [17]. This multicenter radiological research network was initiated during the COVID pandemic and is a joint project of radiology departments at all 38 German university hospitals to annotate a large number of lung CT scans in a structured and uniform manner. Further, this study is the first in the worldwide that uses SATORI in rheumatology to evaluate ILD related to IRD.

The aim of this study was to evaluate the intra-reader reliability of SATORI in terms of AI-based detection and quantification of typical radiological features (like GGO, reticulations, granuloma) in typical HRCT images in IRD-ILD patients (pure GGO, NSIP, UIP, granulomatous disease) to enable more reliable, accurate, and efficient clinical decisions. The aim of our study is therefore not the classification of the CT pattern, but the quantification of typical radiological features in IRD and the evaluation of AIqpHRCT in a clinical setting.

## Patients and methods

To evaluate the AIqpHRCT, our investigation was divided into an experimental part (I) and a clinical part (II).

I. Experimental study

**Fig. 1:**
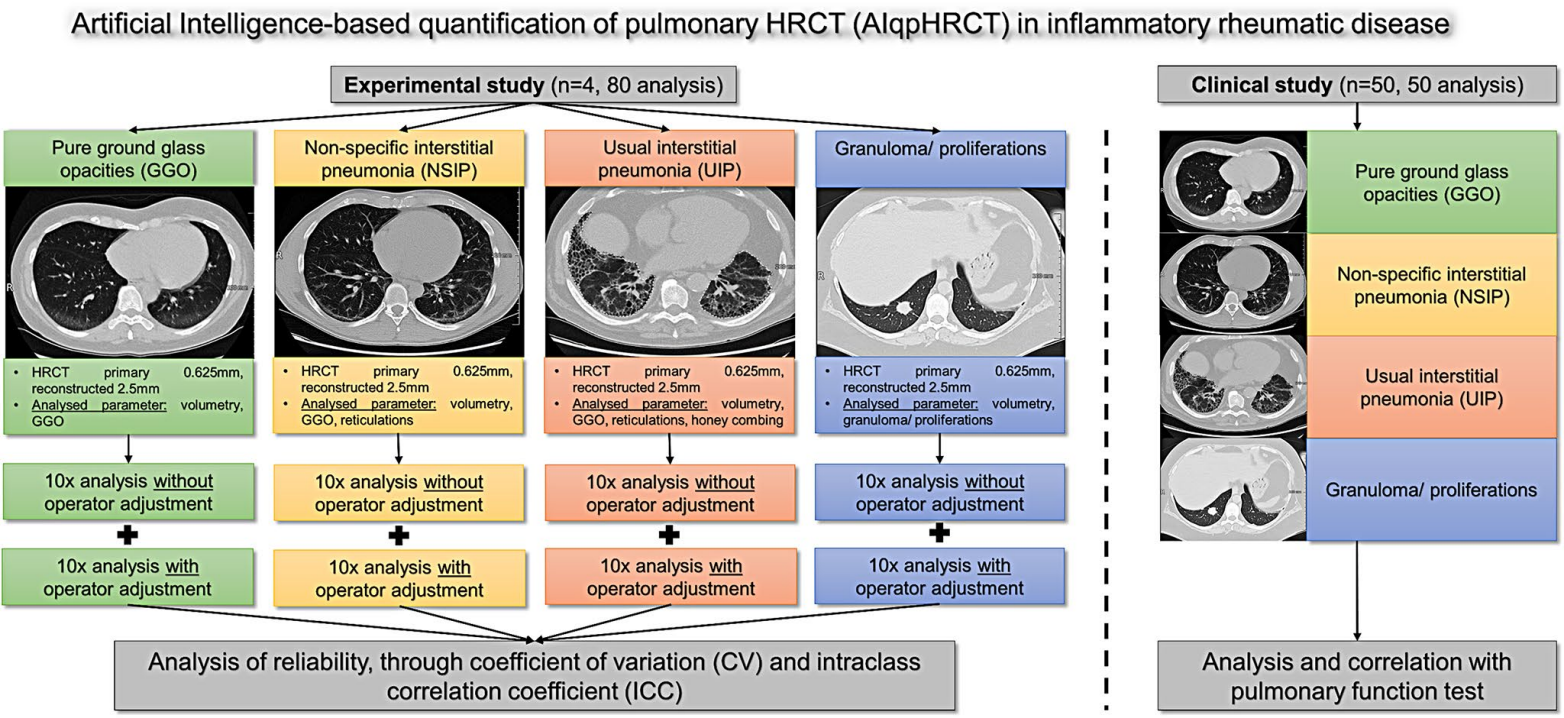
Patients, methods, and study protocol of the experimental and clinical part of the study

**Fig. 2:**
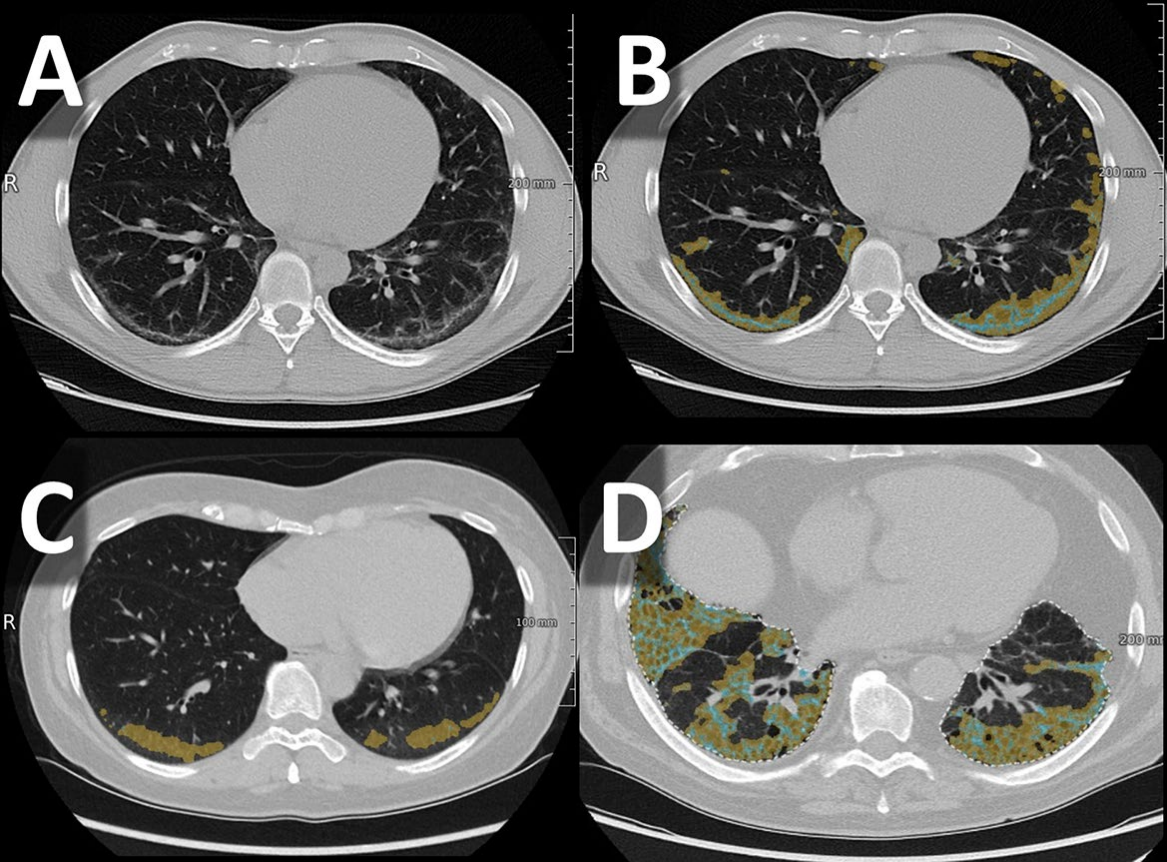
Different high-resolution computed tomography (HRCT) patterns with AI-based segmentation of pulmonary HRCT using SATORI; **A** – Non-specific interstitial pneumonia without AI-based segmentation; **B** – Non-specific interstitial pneumonia; **C** – Ground glass opacities; **D** – Usual interstitial pneumonia

Within the experimental study, 80 HRCT datasets of four immunosuppressant-naive patients (4 patients x 10-fold measurement without and with operator adjustment) with different IRD-ILD were analysed using AI-based quantification with the SATORI platform (AI-based quantification of pulmonary HRCT; AIqpHRCT]) (see study protocol in Fig. 1; Table 1; Fig. 2). These four patients were randomly selected from clinical routine for typical HRCT patterns. All HRCT were performed as part of organ screening for lung involvement for clinical routine and not for study purposes.

**Table 1:** Experimental study – baseline characteristics of the analysed patients

	Patient 1	Patient 2	Patient 3	Patient 4
**Gender**	female	male	male	female
**Age**	40 years	37 years	80 years	36 years
**Disease**	Mixed connective tissue disease	Dermatomyositis	Systemic lupus erythematosus	Granulomatosis with polyangiitis
**Disease duration**	7 months	Initial diagnosis	Initial diagnosis	Initial diagnosis
**Symptom duration**	19 months	New	20 months	New
**HRCT pattern**	Ground-glass opacity (GGO)	Non-specific interstitial pneumonia (NSIP)	Usual interstitial pneumonia (UIP)	Granuloma/ proliferations
**Immunology laboratory**	Antinuclear antibody > 1:1280 Anti-RNP/Sm Anti-SSA	Antinuclear antibody = 1:1280 Anti-PM-Scl	Antinuclear antibody = 1:320 Anti- double-stranded DNA	Anti-neutrophil cytoplasmatic antibody = 1:320 Anti-proteinase-3
**Other organ involvements**	Cardiac involvement	-	-	Cardiac involvement Ophthalmologic involvement Dermal involvement Joint involvement
**Symptoms**	Dyspnoea	No symptoms	Dyspnoea, sclerosiphonia	No symptoms

II. Clinical study.

As part of the clinical study, 50 HRCT datasets from patients with various IRD-ILD with or without immunosuppressive therapy were analysed using AI-based quantification (AIqpHRCT) with the SATORI platform. All HRCT were performed as part of clinical routine and not for study purposes. These 50 patients were selected from clinical routine and independently of underlying HRCT pattern or disease. Clinical data (disease, medication, PFT) were also collected (see Table 2).

**Table 2:** Clinical study – baseline characteristics and AIqpHRCT data of 50 patients with inflammatory rheumatic diseases and interstitial lung disease

Item			Ground glass opacities (GGO)	Non specific interstitial pneumonia (NSIP)	Usual interstitial pneumonia (UIP)	Granuloma/ proliferations	*p* value
**Number**			*n* = 10	*n* = 25	*n* = 12	*n* = 3	
**Age in years** (median [minimum - maximimum])			60.0 (39.0–77.0)	57.0 (20.0–82.0)	61.0 (41.0–79.0)	64.0 (29.0–73.0)	*p* = 0.884
**Gender**							*p* = 0.550
	Woman		*n* = 7 (70.0%)	*n* = 18 (72.0%)	*n* = 9 (75.0%)	*n* = 1 (33.3%)	
	Men		*n* = 3 (30.0%)	*n* = 7 (18.0%)	*n* = 3 (25.0%)	*n* = 2 (66.7%)	
**Disease duration in years** (median [minimum - maximimum])			4.1 (2.4–45.8)	4.7 (1.1–25.8)	14.5 (2.7–42.4)	8.9 (6.8–11.2)	*p* = 0.007
**Immunosuppressive therapy**							*p* < 0.001
	csDMARD		*n* = 9	*n* = 19	*n* = 11	*n* = 1	
	bDMARD		*n* = 0	*n* = 1	*n* = 0	*n* = 2	
	no medication (except prednisolone)		*n* = 1	*n* = 5	*n* = 1	*n* = 0	
**Disease**							*p* < 0.001
	Collagenosis		*n* = 8	*n* = 16	*n* = 11	*n* = 0	
	Myositis		*n* = 1	*n* = 8	*n* = 1	*n* = 0	
	Rheumatoid arthritis		*n* = 1	*n* = 0	*n* = 0	*n* = 0	
	Vasculitis		*n* = 0	*n* = 1	*n* = 0	*n* = 3	
**Pulmonary function test in%** (median [minimum - maximimum])							
	Forced expiratory volume per second (FEV1)		85.3 (51.4–116.2)	79.0 (44.4–110.2)	70.9 (45.8–85.6)	86.1 (47.9–87.0)	*p* = 0.150
	Forced vital capacity (FVC)		77.3 (43.8–102.9)	75.3 (45.9–120.4)	61.1 (50.7–81.1)	77.0 (67.7–98.1)	*p* = 0.181
	Total lung capacity (TLC)		88.0 (48.8–108.5)	70.7 (50.6–101.7)	70.2 (41.4–95.5)	101.9 (78.6–106.0)	*p* = 0.013
	Diffusing capacity of the lung for carbon monoxide (DLCO)		66.4 (35.8–85.1)	56.1 (29.5–92.2)	42.8 (19.3–69.6)	90.9 (84.7–102.3)	*p* = 0.007
**AIqpHRCT data** (mean value ± standard deviationand as wells as median [minimum - maximimum])							
	Volumetric in liter		4.1 ± 1.4 4.1 (2.1–7.3)	3.6 ± 1.0 3.4 (2.2–5.4)	3.3 ± 0.6 3.3 (2.2–4.3)	5.2 ± 0.7 5.1 (4.6–6.0)	*p* = 0.049
	High attenuated volume (HAV) in %		13.1 ± 21.7 6.5 (4.4–74.8)	12.4 ± 8.1 8.8 (5.1–33.1)	14.0 ± 6.5 13.6 (6.2–27.5)	6.0 ± 1.5 5.9 (4.6–7.5)	*p* = 0.012
	Overall extent in %		10.7 ± 28.3 1.3 (0.1–91.0)	13.8 ± 17.6 6.0 (0.9–80.4)	18.9 ± 12.4 18.0 (2.7–37.0)	0.9 ± 0.8 0.8 (0.2–1.8)	*p* < 0.001
		Ground glass opacities (GGO) in %	4.6 ± 9.7 1.1 (0.1–32.1)	9.8 ± 13.7 5.1 (0.8–66.3)	8.7 ± 6.2 7.2 (2.0–25.1)	0.6 ± 0.4 0.7 (0.1–1.0)	*p* = 0.001
		Reticulations in %	6.0 ± 18.4 0.2 (0.0–58.4)	3.8 ± 5.2 1.3 (0.1–17.3)	3.8 ± 3.4 3.1 (0.3–11.9)	0.2 ± 0.2 0.1 (0.0–0.3)	*p* = 0.001
		Honey combing in %	0.1 ± 0.2 0.0 (0.0–0.6)	0.2 ± 0.5 0.0 (0.0–2.2)	6.4 ± 7.4 3.5 (0.0–23.1)	0.0 ± 0.0 0.0 (0.0–0.0)	*p* < 0.001
		Granuloma/ proliferations in %	0.0 ± 0.0 0.0 (0.0–0.0)	0.0 ± 0.1 0.0 (0.0–0.4)	0.0 ± 0.0 0.0 (0.0–0.5)	0.2 ± 0.2 0.1 (0.1–0.5)	*p* = 0.051

The following inclusion criteria were met:

1. The IRD-diagnosis was performed by a comprehensive rheumatologic assessment including clinical, laboratory and imaging data under consideration of rheumatic diagnostic and classification criteria [18].
2. ILD in IRD was diagnosed by a multidisciplinary consensus panel of rheumatologist, pulmonologist and radiologist.
3. HRCT that fulfils the criteria listed below (see section HRCT).
4. Availability of a pulmonary function test at the time of HRCT, with forced expiratory volume per second (FEV1), forced vital capacity (FVC), total lung capacity (TLC) and diffusing capacity of the lung for carbon monoxide (DLCO).
5. Complete data sets concerning baseline characteristics of IRD (disease duration and immunosuppressive therapy).

### HRCT

Multi-slice computed tomography was used for all HRCT images (General Electric Healthcare Technologies, Revolution, Waukesha, Wisconsin; USA) with a primary slice thickness of 0.625 mm and an overlapping reconstructed multiplanar slice thickness of 2.5 mm. The radiation dose was 78,93 mGy*cm. All scans were reviewed in consensus (*qualitative analysis*) by two chest radiologists and one rheumatologist regarding HRCT patterns (GGO, NSIP, UIP and granulomas/proliferations) according to the American Thoracic Society/European Respiratory Society and Fleischner Society White Paper recommendations/criteria. [5–7].

We performed a *quantitative analysis* of the most common pulmonary parenchymal ILD-features in HRCT (each slice of all scans) using SATORI by a rheumatologist. SATORI was trained using supervised machine learning from HRCT, which was developed during the COVID pandemic.

According to international recommendations/criteria and available publications, the characteristic changes are:

1. Pure GGO at early stage of IRD-ILD [3, 19, 20].
2. NSIP with the radiographic features of GGO and reticulation [5–7].
3. UIP with the radiographic features of GGO, reticulation, and honeycombing [5–7].
4. Granuloma/proliferations in granulomatous disease [21–23].

Since SATORI does not allow automated quantification of honeycombing and traction bronchiectasis, a manual segmentation was carried out to capture honeycombing.

SATORI provides automatic and fast segmentation of the lung and lobes based on a 3D U-net with an optimised loss function that focuses on the lobar boundaries [24]. This volumetry (in ml) is the basis for calculating the relative proportions of pathological lung changes. All SATORI examinations were performed using a primary slice thickness of 0.625 mm and a reconstructed and analysed slice thickness of 2.5 mm, which is the standardized thickness for AIqpHRCT.

### Workflow

The HRCT images were obtained from the hospitals picture archiving and communication system (PACS) and pseudonymized using an in-house developed DICOM pseudonymization platform before transferring into the RACOON infrastructure. Afterwards, the corresponding HRCT images were accessed via the web-based SATORI interface [25] and lung parenchymal changes were quantified. These quantitative data were then extracted from SATORI on an analysis/case basis and converted into an Excel file for further analysis.

### Study protocol

The experimental study protocol included the following steps:

1. **AIqpHRCT without operator adjustment**: Evaluation of reproducibility of the volumetry (in ml) and detection of GGO, reticulation, and granuloma/proliferation. Reproducibility was verified by ten repeated analyses of the HRCT of the four patients with GGO, NSIP, UIP, and granuloma.
2. **AIqpHRCT with operator adjustment**: Tenfold adjustment of the size of the volumetry (in ml), GGO, reticulation, and granuloma/proliferation by SATORI, followed by an operator adjustment after each evaluation. Subsequently, a quantification of the reproducibility was carried out.

The clinical study protocol included the following analysis steps:

**Adapted AIqpHRCT**: Measurement of the following values *without operator adjustment*: Volumetry (in ml), GGO, reticulation, and granuloma/proliferation. Measurement of the following values *with operator adjustment*: Honey combing.

### Data analysis

Statistical analysis was performed using Microsoft^Ⓡ^ Excel (Microsoft Windows, Redmond Washington, USA) and IBM SPSS Statistics 28 (IBM SPSS Statistics, Chicago, Illinois, USA, for Windows). P-value < 0.05 was considered as statistically significant. Further, the data analysis was divided regarding the experimental and clinical study.

I. Experimental study

For statistical analysis, results were expressed as median with minimum and maximum (min. – max.) and precision errors as coefficient of variation (CV) and intraclass correlation coefficient (ICC) with 95% confidence interval. The coefficients of variation were given on a percentage basis: CV (in %) = (standard deviation/mean) x 100. In the analysis of relative difference between AIqpHRCT without and with operator adjustment, operator adjustment was considered as the reference/gold standard. Potential outliners were not excluded from the analysis.

II. Clinical study

For statistical analysis, results were expressed as median with minimum and maximum (min. – max.), also mean value with standard deviation in AIqpHRCT data and group differences were analysed using the chi-squared test or Kruskal-Wallis test with a post-hoc test (Dunn’s test). Spearman correlation (ρ) with 95% confidence interval was used to assess the correlation between pulmonary function parameters and AIqpHRCT data, given the expected non-linear relationship between AIqpHRCT data and pulmonary function parameters. Due to the expected loss of lung function with increasing ILD, only 1-sided significance was reported. With regard to Cohen, |ρ| = 0.1 to 0.3 demonstrate a weak, |ρ| = 0.3 to 0.5 a moderate and |ρ| > 0.5 a strong correlation [26]. The graphical visualization was carried out using box plots.

To assess the study’s power given the sample size (*n* = 50), a post-hoc power analysis was conducted. Employing a monte carlo simulation (10.000 times), we determined the statistical power for detecting one-sided Spearman rank correlations across different expected correlation coefficients ρ (weak: >=0.1, moderate > = 0.3, strong: >=0.5) using a significance level of α = 0.05.

Potential outliners were not excluded from the analysis.

## Results

I. **Experimental study**

### Reproducibility of AIqpHRCT in detecting IRD-ILD without operator adjustment

AIqpHRCT without operator adjustment resulted in 100% agreement of the measured values (CV = 0.00% and ICC = 1.000; 95%CI 1.000–1.000) for GGO, NSIP (GGO and reticulation), UIP (GGO and reticulation without honeycombing) as well as granuloma/proliferation.

### Reproducibility of AIqpHRCT in detecting IRD-ILD with operator adjustment

#### Volumetry

Regarding the corresponding HRCT pattern, the mean reliability for the volumetry ranged between CV = 0.33% in GGO and CV = 2.31% in UIP, both with ICC = 1.000 (95%CI 1.000–1.000) in all patterns. In detail, the reliability for lung lobes could be calculated with the following ranges: CV = 0.10% (left upper lobe in granuloma) to CV = 8.52% (right middle lobe in UIP). Considering all analyses, a reliability with a median CV = 0.48% (0.04–8.52%) and ICC = 1.000 (95%CI 1.000–1.000) could be achieved by operator-adjusted measurements of the volumes in IRD-ILD patients.

#### Ground-glass opacity (GGO)

We evaluated the following CV values in respect of GGO ranged between CV = 9.25% (right lower lobe) and CV = 47.10% (left upper lobe) with an ICC = 0.998 (95%CI 0.996-1.000) (Fig. 2C).

#### Non-specific interstitial pneumonia (NSIP)

Concerning GGO as a component of NSIP, the CV ranged from 4.92% (left lower lobe) to 25.54% (right upper lobe) with an ICC = 0.998 (95%CI 0.993-1.000). Lower CV values were presented for the reticulation component ranging between 1.02% (right lower lobe) and 12.48% (right middle lobe) with an ICC = 1.000 (95%CI 1.000–1.000) (Fig. 2A and B).

#### Usual interstitial pneumonia (UIP)

The CV values for GGO as a component of UIP ranged from 3.12% (right lower lobe) to 10.50% (right upper lobe) with an ICC = 1.000 (95%CI 0.999-1.000). For reticulation, CV values ranged from 0.001% (right upper lobe) to 15.89% (right middle lobe) with an ICC = 0.999 (95%CI 0.997-1.000). Regarding honeycombing, a CV between 4.20% (left lower lobe) and 26.91% (right middle lobe), with an ICC = 0.997 (95%CI 0.992–0.999) was observed (Fig. 2D).

#### Granuloma/proliferation

For granuloma/proliferation the CV ranged between 0.001% (left lower lobe) to 28.75% (left upper lobe) with an ICC = 0.999 (95%CI 0.999-1.000) (Fig. 1).

#### Mean CV regarding pathological changes

For pathological changes on HRCT, the following reliabilities were observed: GGO – median CV = 9.23% (0.00–47.10%), mean ICC = 0.988 (95%CI 0.979–0.995); reticulation – median CV = 5.36% (0.00–15.89%), mean ICC = 0.997 (95%CI 0.994–0.999); granuloma/proliferation – median CV = 0.00% (0.00–28.75%), mean ICC = 0.994 (95%CI 0.985–0.999); honeycombing – median CV = 6.43% (0.00–26.91), mean ICC = 0.997 (95%CI 0.992–0.999).

### Differences in AIqpHRCT with and without operator adjustment

For GGO, the difference between AIqpHRCT with and without operator adjustment was 39.25%. Regarding NSIP, a difference of 20.33% (GGO component) and 1.49% (reticulation component) was observed. For UIP, the following differences were evaluated: 4.20% (GGO component) and 0.05% (reticulation component). A difference of 0.68% was revealed between AIqpHRCT with and without operator adjustment for granulomas.

II. **Clinical study**

The clinical part of this study encompassed 50 from clinical routine selected patients with ILD in IRD. The underlying HRCT pattern were NSIP (*n* = 25, 50.0%), UIP (*n* = 12, 24.0%), pure GGO (*n* = 10, 20.0%), and 3 patients (6.0%) with known small vessel vasculitis (granuloma/ proliferations). There were no significant differences in baseline characteristics with respect to age or gender, but there were significant differences in disease distribution or use of immunosuppressive drugs (determined in the group of patients with granuloma; *n* = 3) at the time of HRCT performance. There was also a significant difference in disease duration (patients with UIP having a longer disease duration).

### Pulmonary function test

**Fig. 3:**
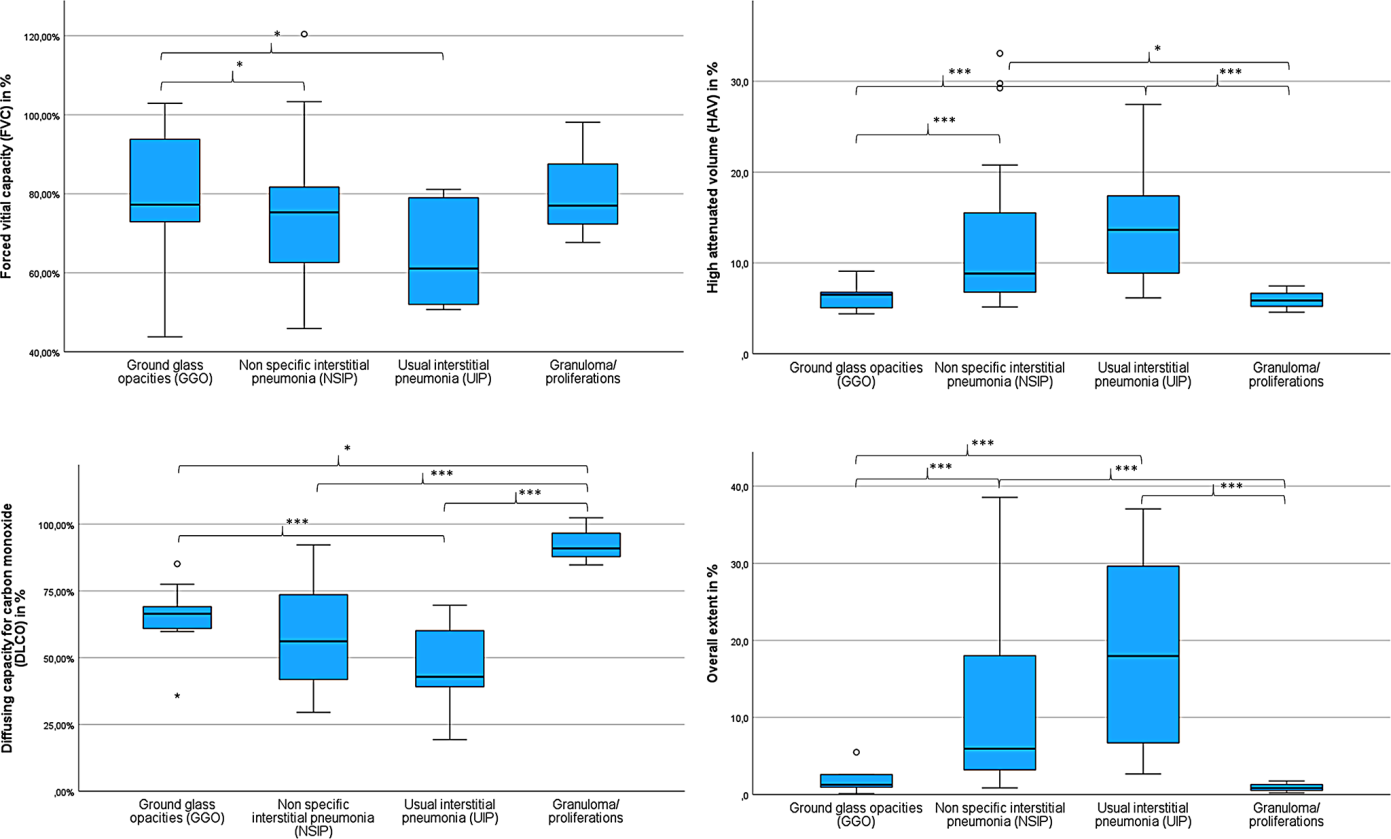
Pulmonary function test and measured AIqpHRCT data in patients with ILD (GGO, NSIP, UIP and granuloma) – L*eft column*: Pulmonray function parameters with FVC and DLCO, *right column*: AIqpHRCT data with HAV and overall extent (***=*p* < 0.05, *=*p* < 0.1)

The analysis of pulmonary lung function parameters in patients with GGO, NSIP, and UIP pattern revealed the following median values for FVC: GGO 77.3% (43.8–102.9%), NSIP 75.3% (45.9–120.4%) and UIP 61.1% (50.7–81.1%) as well as for DLCO: GGO 66.4% (35.8–85.1%), NSIP 56.1% (29.5–92.2%) and UIP 42.8% (19.3–69.6%). This progressive loss of lung function was also reproducible in TLC and FEV1 in the analysed HRCT pattern. Therefore, the analysis showed significant differences in TLC between GGO – NSIP (*p* = 0.027), GGO – UIP (*p* = 0.02), but also in DLCO between GGO – UIP (*p* = 0.031) (see Table 2; Fig. 3).

Analysis of PFT in patients with small vessel vasculitis showed near-normal pulmonary function parameters, with median values > 80% predicted in TLC, FVC, and DLCO. (see Table 2; Fig. 3)

### AIqpHRCT data

AIqpHRCT data were measured in all 50 ILD-IRD patients. With regard to significant difference in volumetry (*p* = 0.049), relative values in % have been used. All analysed pulmonary pathologies revealed significant differences between the groups (see Table 2).

The analysis of patients with pure GGO showed 4.6 ± 9.7% (median 1.1%) GGO, 6.0 ± 18.4% (median 0.2%) reticulations and 0.1 ± 0.2% (median 0.0%) honey combing, with an overall extent of 10.7 ± 28.3% (median 1.3%). In patients with NSIP 9.8 ± 13.7% (median 5.1%) GGO, 3.8 ± 5.2% (median 1.3%) reticulations and 0.2 ± 0.5% (median 0.0%) honey combing were measured, with an overall extent of 13.8 ± 17.6% (median 6.0%). Furthermore, AIqpHRCT revealed 8.7 ± 6.2% (median 7.2%) of GGO, 3.8 ± 3.4% (median 3.1%) of reticulations and 6.4 ± 7.4% (median 3.5%) of honey combing, with an overall extent of 18.9 ± 12.4% (median 18.0%) in UIP pattern. The analysis demonstrated significant differences between GGO – NSIP and GGO – UIP at the amount of GGO (in %), as well as at the overall extent (in %) (see Table 2; Fig. 3).

Measured pulmonary pathologies through HAV revealed 13.1 ± 21.7% (median 6.5%) in GGO, 12.4 ± 8.1% (median 8.8%) in NSIP, and 14.0 ± 6.5% (median 13.6%) in UIP patients, showing also significant differences between these groups (*p* = 0.012) (see Table 2; Fig. 3).

### Correlation of pulmonary function test and AIqpHRCT data

For clinical evaluation of AIqpHRCT, correlation with PFT as surrogate parameters in ILD was performed. The volumetry of AIqpHRCT demonstrated a correlation of ρ= +0.543 (95%CI 0.352 to 1.000) and ρ= +0.540 (95%CI 0.348 to 1.000) with FVC and TLC (*p* < 0.001). Furthermore, there was a similar negative correlation of FVC (ρ= -0.501; 95%CI -1.000 to -0.300), TLC (ρ= -0.622; 95%CI -1.000 to -0.452) and DLCO (ρ= -0.693; 95%CI -1.000 to -0.546) with the overall extent (*p* < 0.001), but also HAV with FVC: ρ= -0,531 (95%CI -1.000 to -0.377), TLC: ρ= -0,657 (95%CI -1.000 to -0.498) and DLCO: ρ= -0,629 (95%CI -1.000 to -0.461) with *p* < 0.001 (see Table 3; Fig. 3).

**Table 3:** Spearman correlation with 95% confidence interval (CI) and power analysis of measured AIqpHRCT data with lung function parameters

Spearman correlation (ρ)		FEV1 in %	FVC in %	TLC in %	DLCO in %
**Volumetric** in liter (95% CI)		0.379 (0.157, 1.000)	0.543 (0.352, 1.000)	0.540 (0.348, 1.000)	0.278 (0.045, 1.000)
	p-value (1-sided)	0.003	< 0.001	< 0.001	0.029
	power analysis	0.864	0.994	0.993	0.625
**High attenuated volume (HAV)** in % (95% CI)		-0.491 (-1.000, -0.337)	-0.531 (-1.000, -0.377)	-0.657 (-1.000, -0.498)	-0.629 (-1.000, -0.461)
	p-value (1-sided)	< 0.001	< 0.001	< 0.001	< 0.001
	power analysis	0.979	0.991	1.000	1.000
**Overall extent** in % (95% CI)		-0.482 (-1.000, -0.277)	-0.501 (-1.000, -0.300)	-0.622 (-1.000, -0.452)	-0.693 (-1.000, -0.546)
	p-value (1-sided)	< 0.001	< 0.001	< 0.001	< 0.001
	power analysis	0.974	0.983	0.999	1.000
**Ground glass opacities (GGO)** in % (95% CI)		-0.391 (-1.000, -0.171)	-0.423 (-1.000, -0.208)	-0.580 (-1.000, -0.398)	-0.699 (-1.000, -0.554)
	p-value (1-sided)	0.002	0.001	< 0.001	< 0.001
	power analysis	0.884	0.927	0.998	1.000
**Reticulations** in % (95% CI)		-0.536 (-1.000, -0.343)	-0.533 (-1.000, -0.340)	-0.655 (-1.000, -0.496)	-0.643 (-1.000, -0.480)
	p-value (1-sided)	< 0.001	< 0.001	< 0.001	< 0.001
	power analysis	0.992	0.991	1.000	1.000

With regard to measured GGO in AIpqHRCT, analysis demonstrated a negative correlation with DLCO (ρ= -0,699; 95%CI -1.000 to -0.554; *p* < 0.001), TLC (ρ= -0,580; 95%CI -1.000 to -0.398; *p* < 0.001), and FVC (ρ= -0,423; 95%CI -1.000 to -0.208; *p* = 0.001) (see Table 3; Fig. 3).

High power (range: 0.625-1.000) was shown by strong correlations, indicating a high probability of detecting a strong effect (see Table 3).

## Discussion

We performed a quantitative analysis of the most common pulmonary parenchymal ILD-features in HRCT in IRD-patients, as well as a clinical trial with 50 ILD-IRD patients, using AIqpHRCT based on the browser-based toolkit SATORI. This system provides a workflow for quantitative lung parenchyma analysis including AI-based fast segmentation of lungs and lung lobes. It should be noted that a validation of AI analysis for ILD in IRD does not exist.

### Experimental study

Handa et al. demonstrated a moderate to strong correlation between AI-based analysis and visual scoring in IPF-patients depending on the parenchymal pattern [27]. In addition, Wu et al. showed a numerical difference between AI (14.1 ± 11.30) and observer-based analysis/scoring (24.5 ± 13.8) in IPF, without analysing the significance [28].

About ICC, our measurements showed an excellent reliability in the AIqpHRCT segmentation without operator adjustment (ICC = 1.000). According to Koo and Li, an ICC of > 0.9 is classified as excellent [29]. In our study, the reproducibility of AIqpHRCT with operator adjustment for all pathological changes of IRD-ILD (GGO – ICC = 0.988, reticulation – ICC = 0.997, granuloma – ICC = 0.994 and honeycombing – ICC = 0.997) in HRCT was also very high.

The correct detection and segmentation of the lungs and lung lobes are essential for a high reliability, especially in patients with increased fibrosis (NSIP, UIP), but also in patients with pleural proliferation (granuloma/proliferation). SATORI uses an AI-based segmentation of the lung lobes, based on fissures, vessels, and bronchi [30, 31]. Lassen-Schmidt et al. demonstrated, that both the interactive corrections and the creation of a segmentation from scratch using SATORI led to excellent results and only little interaction, even in patients with severe pathologies [30, 32].

Nevertheless, there is a significant intra-individual difference in CV, which is important for analysis and the obtained results based on AI analysis with operator adjustment. In GGO, the analysed median CV = 9.23% (0.00–47.10%), resulting in an intra-individual difference in the measurement of GGO. This is mainly due to the transition from GGO to healthy lung tissue, which cannot be clearly distinguished and is different from COVID-19 pneumonia with acute inflammation [10, 12, 33]. For the other fibrosis-associated pulmonal changes (reticulation and honeycombing), similar median CV between 5.36% and 6.43% could be obtained. In contrast, AIqpHRCT without operator adjustment showed excellent reproducibility (CV < 0.001), so that repeated AIqpHRCT analysis led to the same results.

In summary, the study reports excellent reproducibility for AIqpHRCT without operator adjustment, the reproducibility significantly decreases when operator adjustments are introduced, especially for features like GGO and honeycombing which is based on the intra-individual variability with operator adjustments. This variability undermines the reliability of AIqpHRCT with operator adjustments in clinical practice and for longitudinal studies where consistent measurements are critical. Consequently, in clinical studies as well as in clinical practice, only the quantification of IRD related ILD by AIqpHRCT without operator adjustment can be recommended in order to obtain consistent measurements. The value of AIqpHRCT with operator adjustment has to be evaluated in further studies.

### Clinical study

In the clinical part of this study, we analysed a group of patients with ILD in IRD selected out of clinical routine (*n* = 50). As expected, in a group of patients receiving adequate immunosuppressive therapy, there was a high proportion of NSIP patterns (*n* = 25, 50.0%), but also UIP (*n* = 12, 24.0%) and GGO (*n* = 10, 20.0%) patterns. Regarding the included diseases with collagenosis (70.0%), myositis (20.0%), rheumatoid arthritis (2.0%) and vasculitis (8.0%), this study representing a typical cohort of IRD-ILD patients, as demonstrated in previous studies [3, 34]. Moreover, collagenosis are the main causes of ILD in rheumatology alongside myositis [35].

Pulmonary function testing, a well known and extensively evaluated surrogate parameters in ILD, showed typical changes with progressive loss of lung function in progressive ILD (from GGO to NSIP and UIP) in all measured parameters (FEV1, FVC, TLC and DLCO) [36, 37]. As reported in the literature, patients with moderate ILD (pure GGO) also showed a typical loss of DLCO in contrast to the other measured lung function parameters [2, 3]. In contrast to fibrotic ILD, there was no significant variation in PFT compared to the reference of > 80% predicted in patients with small vessel vasculitis (granuloma/proliferation) in our study, also described in the literature [3].

Regarding the analysed AIqpHRCT data, Le Gall et al. described an AI-based analysis for HRCT in SSc, where ILD could only be considered as a total extent, presenting an ILD extent of 11.0 to 26.6% with a median disease duration of 6 years [38].

To the best of our knowledge, there is currently no AI-based software that is able to analyse ILD in detail (GGO, reticulation, honeycombing, granuloma). Palmucci et al. and Kaya et al. demonstrated similar correlations coefficients of -0.358 to -0.564 for FVC and − 0.451 to -0.750 for DLCO to our study, in patients with ILD from various causes (autoimmune, but also idiopathic), examined through a histogram/kurtosis-based analysis (comparable to HAV) [39, 40]. However, by using AIqpHRCT it is also possible to analyse GGO, reticulations, granuloma, and honeycombing. Our data revealed on the one hand the highest correlation of GGO with DLCO (ρ=-0.699), but on the other hand also a high correlation of TLC with fibrotic changes (reticulations) (ρ=-0.655). By differentiating the pathologies (GGO and reticulations) through AIqpHRCT, the effect on the PFT can be clarified and thus the correlation coefficients shown with a clinically appropriate strong inverse correlation (ρ > 0.5) of the pulmonary function parameters to the extent of ILD. Therefore, the extent of the reduced pulmonary function parameters is directly associated with the affected lung tissue by ILD. Consequently, these patients may require more intensive therapy for IRD-associated ILD.

In the future, early detection of pulmonary involvement in IRD will become even more important. This is due to the fact that lung manifestations are increasingly recognized to be a major complication and a risk factor for a poor prognosis in these patients. In this context, it will become important that ILD is quantified, e.g. through AIqpHRCT, as part of the initial diagnostic of IRD related ILD, as minimal radiographic progression of > 2% in SSc over 12/24 months predict increased risk for long-term mortality [41]. Further, based on immunological bronchoalveolar lavage analysis, GGO in HRCT represented immunological inflammation of the alveoli and NSIP as well as UIP were associated with inflammation and/or fibrosis of the lung tissue [34]. Consequently, AIqpHRCT is able to quantify immunological inflamed (GGO) and inflammatory/fibrotic lung tissue (reticulation/ honey combing in NSIP or UIP) which is very important concerning the therapy strategy with a primary anti-inflammatory or anti-fibrotic treatment or a combination of both. In addition, new therapeutic targets are finding their way into the treatment of IRD-ILD. Therefore, AIqpHRCT may further support precise monitoring of therapy response.

Our study demonstrated a high reliability and reproducibility of AIqpHRCT using the browser-based platform SATORI for detecting parenchymal changes in IRD-ILD-patients without operator adjustment. Moreover, we demonstrated a high invers correlation to established surrogate parameters in ILD, like FVC for mortality or DLCO for screening [3, 42]. This is a critical finding because AIpqHRCT with SATORI could be used not only to assess lung parenchymal abnormalities in IRD-ILD, but also to precise monitor therapy with new anti-inflammatory and anti-fibrotic drugs [43].

The absence of an established scoring system for quantifying IRD-ILD using AIqpHRCT substantial limits the study’s ability to benchmark its findings against current gold standards. This gap could be addressed, either through the development of a new scoring system or by comparing the AI-generated data to existing clinical assessments. Further, the study represented an initial feasibility study and the sample size is limited with a potential restriction of the generalizability of the findings. To evaluate the generalizability of the data, power analysis was performed, showing high power (> 0.8) for all measured ILD features (GGO, reticulations, HAV and overall extent). As high power (> 0.8) was shown by strong correlations, indicating a high probability of detecting a strong effect, the study demonstrated sufficient statistical power to reliably detect moderate and strong correlations. Based on the promising data, these initial findings should be verified in a large multicentre study to investigate this method, including clinical parameters.

## Conclusion

In conclusion, our study demonstrated an excellent reliability for AIqpHRCT using the browser-based platform SATORI in IRD-ILD analysis without and with operator adjusted measurement, defined by the ICC. However, an intra-individual variation between 5% and 10% of the measured values of parenchymal changes such as GGO, reticulation and granuloma was observed using AIqpHRCT with operator-adjusted measurements. If AlqpHRCT is applied to detect lung changes in IRD-ILD, this should be done without operator adjustment in the majority of clinical settings. Moreover, AIqpHRCT data demonstrated a high negative correlation to surrogate parameters in PFT. Here, the correlation of GGO with DLCO and reticulations and total extent of ILD with FVC and TLC is highlighted.

We have shown for the first time, that an AI-based analysis with SATORI allows the quantification of parenchymal changes in IRD-ILD-patients. In the future, we will examine the extent of lung involvement and detect therapy responses using SATORI.

## Electronic supplementary material

Below is the link to the electronic supplementary material.

Supplementary Material 1

## Data Availability

Data pertaining to this study is available with the corresponding author and will be shared on reasonable request.

## Abbreviations

AI: Artificial intelligence
AIqpHRCT: AI-based quantification of pulmonary HRCT
CV: Coefficient of variation
CI: Confidence interval
EGPA: Eosinophilic granulomatosis with polyangiitis
GGO: Ground-glass opacity
GPA: Granulomatosis with polyangiitis
HAV: High attenuated volume
HRCT: High-resolution computed tomography
ICC: Intra-class correlation coefficient
ILD: Interstitial lung disease
IRD: Inflammatory rheumatic diseases
MCTD: Mixed connective tissue disease
MPA: Microscopic polyangiitis
NSIP: Non-specific interstitial pneumonia
PACS: Picture Archiving and Communication System
PFT: Pulmonary function test
RACOON: Radiological Cooperative Network
SATORI: Segmentation and Annotation Tool for Radiomics and Deep Learning
SD: Standard deviation
SLE: Systemic lupus erythematosus
SSc: Systemic sclerosis
UIP: Usual interstitial pneumonia
